# Explosive Detection Dogs: A Perspective from the Personality Profile, Selection, Training Methods, Employment, and Performance to Mitigate a Real Threat

**DOI:** 10.3390/ani13243773

**Published:** 2023-12-07

**Authors:** Antônio J. de Miranda-Magalhães, Gustavo M. Jantorno, Adauto Z. Pralon, Márcio B. de Castro, Cristiano Barros de Melo

**Affiliations:** 1 Graduate Program in Animal Sciences (PPGCA/FAV), University of Brasilia (UnB), Campus Darcy Ribeiro ICC Sul, Asa Norte, Brasília 70910-900, Federal District, Brazil; hausmiranda@uol.com.br (A.J.d.M.-M.); gustavojantorno@gmail.com (G.M.J.); mbcastro@unb.br (M.B.d.C.); 2 Bomb and Explosives Group (GBE), Policia Federal (PF/SRES), Brasília 70037-900, Federal District, Brazil; adautoazp@gmail.com

**Keywords:** canine, sniffer dog, terrorism, explosive, detector

## Abstract

**Simple Summary:**

This review analyzes the scientific literature regarding explosive detection dogs (EDDs), focusing on animal personality profile, selection, training methods, employment, and performance. Critical aspects of EDDs were addressed to improve the success of working with dogs in explosive detection and expand knowledge in a very sensitive area. Dissemination of knowledge on the employment and technical training of EDDs is essential to prevent catastrophes caused by explosives and is essential to save lives worldwide.

**Abstract:**

Terrorist organizations have compelled security authorities of every nation to make an increasingly significant commitment toward mitigating the risk of mass casualties and severe financial and property damages. As a result, various security measures have been implemented, including the use of advanced equipment and an uptick in intelligence activities. One of the most effective tools that has yielded outstanding results is the use of explosive detection dogs (EDDs). The nature of EDDs demands a high level of sensitivity given the inherent danger and severity of real threat situations that may involve the risk of explosion. Moreover, the operating procedures for EDDs are unique and distinguishable from other forms of detection. We conducted a review to ensure a comprehensive understanding of the subject, highlighting the EDDs’ personality profile, selection, training methods, performance, and employment, incorporating insights from diverse fields, conducting an analysis, and presenting a perspective on using EDDs to prevent explosion threats.

## 1. Introduction

Detection dogs (DDs) are essential tools for preventing security threats, obtaining evidence, and producing proof of criminal activity. The employment of these dogs is accepted in the courts of justice of various countries, such as England, Wales, Australia, and the United States of America, under strict protocols [1,2,3,4,5,6]. Explosive detection dogs (EDDs) are a unique type of DD that require a multifaceted and multidisciplinary approach, delicate procedures, and a powerful sense of risk to perform their crucial role with high precision and efficiency.

**Figure 1:**
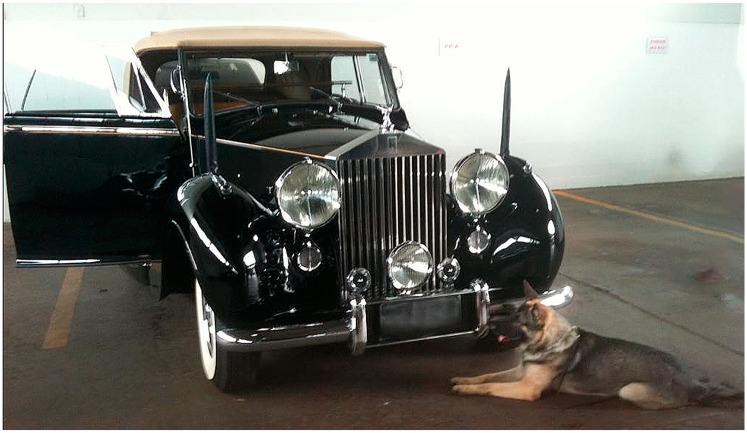
Rolls Royce of the Presidency of the Republic of Brazil being inspected by EDD Kim of the Federal Police during the protection preparations for the inauguration of the President on 1 January 2011. Source: author’s archive.

DDs have their grounds based on knowledge from various scientific fields, such as psychology, veterinary medicine, chemistry, and biology, gathered in law enforcement through canine detection units in their daily work activities [6]. The employment of these dogs includes functions critical to society, such as detecting explosives and bombs [7]. Several security agencies worldwide have established sniffer dog units and have been developing their EDD teams to address the increasing threat posed by terrorist groups. As a result, most sniffer dogs are primarily trained for explosive detection in some countries in response to the global terrorism issue [8]. Figure 1 shows the EDD “Kim” from the Brazilian Federal Police in real action, inspecting the Presidential Rolls Royce during the security inspection protocols for the inauguration of the President of the Republic of Brazil on 1 January 2011.

Individuals acting alone (so-called “lone wolves”), as well as terrorist groups, have cells scattered worldwide with the firm objective of advancing a political goal wherever possible. Authorities, ethnic groups, or significant events can all serve as a target for promoting this objective, putting the lives of tens, hundreds, or thousands of people at risk [9]. To counter this threat, law enforcement agencies in several countries, including the Transportation Security Administration (TSA) in the United States of America (USA), have increased their security measures by incorporating EDD units [10].

Scientists from various universities and research centers have been collaborating with security agencies to enhance the efficiency of using DDs, particularly in the USA [11]. In one such initiative, federal security agencies like the TSA and the Federal Bureau of Investigation (FBI), along with Florida International University (FIU), established the Scientific Working Group on Dogs and Orthogonal Detection Guidelines (SWGDOG) in January 2005. This partnership aimed to develop guidelines based on a consensus that could be implemented by all groups involved in working with DDs [12,13]. However, protocols for EDDs tend to be highly compartmentalized and variable across different EDDs units of law enforcement agencies, with inadequate scientific examination and validation [14]. As a consequence, considering the high risk involved in the work field of EDDs, methodological concerns may give rise to debatable interpretations and lead to divergent results.

Despite the challenging circumstances, taking a deeper look into the subject matter, specifically from the perspective of performance and safety, is crucial when dealing with events that involve real threats and the use of EDDs. It is worth noting that the operational procedures for EDDs differ significantly from other detection areas. Different findings and new questions were formed by analyzing data and information gathered from scientific literature, which allowed valuable insights into enhancing EDD employment, risk mitigation, and better use of public resources.

**Figure 2:**
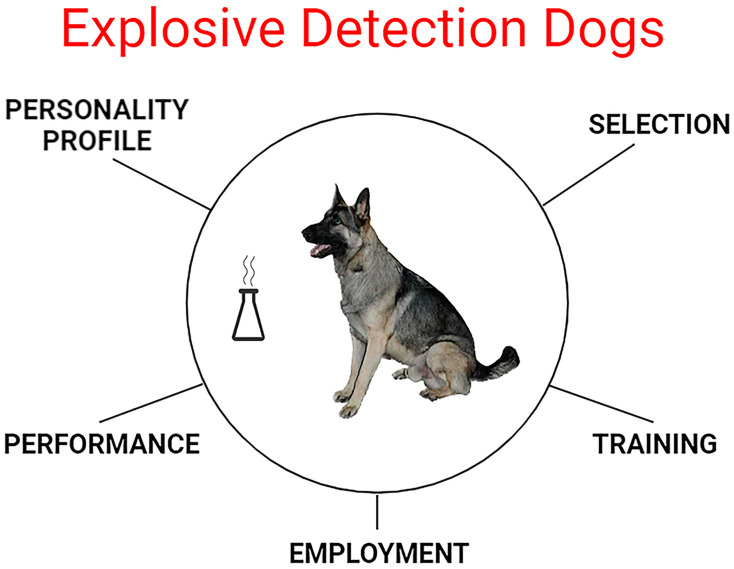
Explosive detection dogs’ requirements for their use in explosion threat mitigation. Source: authors.

This review addresses the personality profile, selection, training, performance, and employment of EDDs (Figure 2) required for an indication of explosive artifacts and effective alternatives to threat mitigation.

## 2. Personality Profile, Selection, and Training Methods of the EDDs

### 2.1. Personality Profile

Setting a personality profile is essential for success in any activity that aims to achieve high performance, such as employing EDDs. The Big Five model (Five Factor Model) was developed to broadly characterize dimensions of the personality as follows: Neuroticism versus Emotional Stability; Agreeableness versus Antagonism; Extraversion versus Introversion; Openness versus Closed to Experience; and Conscientiousness versus Impulsiveness [15]. Regarding personality, dogs are characterized by sociability and energy, and there is evidence of personality differences between dogs [15]. Conscientiousness vs. Impulsiveness (. for example: deliberation, self-discipline, dutifulness, and order) and Openness vs. Closed to Experience (for example: ideas/intellect, imagination, creativity, and curiosity) must be included when evaluating a proper personality profile for EDDs. In terms of personality, dogs show significant differences from humans in levels of Conscientiousness vs. Impulsiveness [15]. In this sense, considering the issue of “Impulsiveness” and given the inherent and significant risk of explosive detection, which requires minimum tolerance of errors, the employment of EDDs with a balanced personality is crucial.

DDs are typically selectively bred based on specific personality traits [16]. Personality is defined as individual differences in behavior correlated in functional contexts over time [16]. Significant variations in personality and behavior have been detected in working dogs since birth, which may influence the success of the work carried out in their adult life. In detection dogs, individual personality differences tend to be prominent, such as fearful behaviors toward strange humans, which can also happen due to distrust in new environments [16].

Important behavioral and physical traits must be identified and taken into consideration during the DD selection process to increase the likelihood of achieving the desired outcomes. Some specific dog breeds are more suitable for the task at hand [16]. In addition to the dogs’ breed, the selection of some anatomical and physiological features of the nose and its size impact the dogs’ ability to detect explosives [17,18,19,20,21]. The length and width of the dog’s nose are directly correlated with the number of olfactory receptors, meaning that breeds with more prominent noses tend to have more receptors [21], and also allow them to retain better and detect a broader range of odors, increasing their olfactory accuracy [17].

In a survey conducted in the United Kingdom (UK) involving dog trainers and handlers, the English Springer Spaniel, Labrador Retriever, and Border Collie were the top three dog breeds used for explosives detection, making up more than 80% of all EDDs [20]. Certain dog breeds, such as sniffer hounds, were intentionally bred for their exceptional ability to hunt and track using olfactory cues, possibly related to their high number of olfactory neurons and elevated sensitivity to odorants [17]. Even though sniffer hounds have an exceptional sense of smell, a number of different breeds have been used as DDs [18]. In practice, the selection of a proper EDD must not be grounded only on anatomical features, such as the number of olfactory neurons and odor receptors.

An alternative approach to selecting EDDs is focused on their behavioral traits. Five behaviors have been detected in different sniffer dog breeds: playfulness, curiosity/fearlessness, chase propensity, sociability, and aggressiveness [22]. Trainability, motivation to sniff, ability to focus on the search and ignore distracting stimuli, temperament, willingness to search without being discouraged by lack of success, and the ability to work effectively in stressful situations are other essential behaviors to be identified in EDDs [23]. A high level of control is crucial for the safety and success of EDDs that operate at a distance from their handlers [14].

Each dog’s behavioral response shows a specific manifestation and level of intensity, which is unique to the individual and can be defined and measured differently [24]. A study conducted at the dog supply and training unit of the US Department of Defense (DoD) at Lackland Air Force Base, Texas, with a significant number of animals (*n* = 1000), showed that behavioral profiles grounded on focus and intensity in search activities in the dog selection process increased the probability of final approval in quality tests [25]. Dogs with remarkable play motivation are able to maintain the focus and interest required for hours of odor target detection [26]. However, an animal with excessive levels of energy and enthusiasm can be detrimental and risky in the task of an EDD [14].

In addition to the behavioral approach, various manifestations, intensity levels, and emotional balance are essential requirements for EDDs. The emotional balance is a combination of innate qualities, life experiences, and training that can either hinder or enhance a dog’s physical and mental development. Working dogs’ ability to adapt and cope with environmental stress is crucial [27], and fear is highly undesirable, especially for EDDs on high-risk missions, which are sometimes exposed to random and unpredictable environmental stimuli [14]. Considering that DDs may be employed in different ways and locations, certain behavioral traits can be or are not essential in the selection of dogs, depending on the purpose.

Drive, agitation, anxiety, excitement, arousal, fear, and stress are terms to describe canine behaviors, but they do not present a standardization in their use. Canine drive is typically considered an enthusiasm, vigor, and willingness to engage in certain behavior that leads to a specific behavior or action [28], which can be influenced by environmental factors and may change over time [17]. According to the American Academy of Forensic Sciences, drive refers to a canine’s inherent propensity to exhibit instinctive behaviors when exposed to certain stimuli, which cannot be created or eliminated [29].

Anxiety is another essential behavior to consider in the selection of EDDs, which refers to a defensive emotional state experienced by the animal when exposed to situations with possible threats, such as entirely new situations or environments that predict an unfavorable outcome [30,31]. Interestingly, higher anxiety levels may be associated with a stronger desire to work and complete detection training [32]. However, the term anxiety in different contexts can lead to contradictory concepts, and the lack of clarity in the definitions of these terms can make it challenging to compare results from various studies. An example is the American Psychological Association (APA) definition of anxiety as an emotion characterized by apprehension and somatic symptoms of tension in which an individual anticipates imminent danger, catastrophe, or misfortune [33,34]. In the canine behavioral evaluation, anxiety is defined as an emotion of apprehension in response to an anticipated danger or threat, and some signs can be recognized, such as autonomic arousal and hypervigilance responses [35].

### 2.2. Training Methods for EDDs

Training includes the methodology essential in determining the personality profile of an EDD, influencing the selection and maintenance of desired behaviors at high levels of efficiency and for an extended period in response to assimilated stimuli processed in the brain to achieve a balance between the individual and the surroundings [36]. In contrast, learning is the process in which an individual modifies their behavior or knowledge through interactions with environmental stimuli [37]. Thus, training programs that align dogs’ cognitive and emotional capacities and account for factors such as repetition, reinforcement, and environmental context are essential to maximize learning efficiency and maintain desirable behavior.

Most canine and EDD training techniques rely on classical and operant conditioning principles [37,38]. A relation between identifying a specific odor and receiving a reward is usually one of the most relevant initial learnings for a DD [27]. Reward is commonly used in detection dog training and is defined by APA as the intention of someone who provides a consequence for behavior rather than the effectiveness of the outcome in influencing the frequency or probability of occurrence of a given behavior [33]. In addition, reinforcement is considered a process in which the frequency or probability of a response is increased by a dependent relationship, or contingency, with a stimulus or circumstance [33]. Food (a biological necessity) or toys (psychological recompense) are commonly used rewards with a high value for the dog as a reinforcing mechanism in search for desired behavior responses in DDs. However, food as a reward may have limitations due to satiety, while the use of toys allows for successive repetitions and more extended work periods for an EDD [14].

Reinforcement is an essential instrument of motivation to continue, inhibit, or even stop a behavior, depending on the standardization of protocols suitable for most dogs, as they have individualized behaviors and learning processes [37]. Considering mistakes can mean many lost lives, a behavior must be well conditioned in EDDs, and the immediate reinforcement tends to increase it, differing from an undesirable behavior which is unreinforced and enabled to decrease or disappear [39]. Low rates of unwanted behavior in DDs have been stated in simple training methods or when exclusively founded on positive reinforcement [23,40,41].

The learning process is complex and involves positive and negative reinforcement and may also include some degrees of controversial positive and negative punishment [42,43,44,45,46,47,48,49]. Reinforcement of desirable behaviors is not the only effective way in EDD training, and the correct application of aversive stimuli, respecting animal welfare, has been proposed to positively propel dogs not to repeat undesirable behaviors, building the capacity to cope with frustration and potential stressors [39,50]. EDD training methods based on positive reinforcement have demonstrated significant advantages over aversive methods and, in addition to the dog trainer’s experience, directly impact the training protocols’ length and success rate [51]. It is essential to highlight that companion dogs trained with aversion-based methods showed worse results during training sessions than dogs trained with reward-based methods [52].

### 2.3. Selection of EDDs

In selective protocols, high performance is defined as a superior characteristic, an ability to operate at a high standard, faster, or more efficiently than others [53,54], and is a *sine qua non* condition for an EDD in real operations. Thus, high performance refers to maximizing an individual’s ability to achieve close-to-perfect outcomes while minimizing the risks of possible failures. Essential traits, such as trainability, self-confidence, focus, resilience, olfactory acuity, precision, and well-being, must be honed to the highest levels in EDDs.

The proper selection of DDs is critical for the success of the intended activity [55] and may be conducted during the dogs’ breeding period or at a young or adult age with the application of specific exercises to identify dogs with desired traits at appropriate levels [14,16,55,56,57,58,59,60,61]. Implementing selection tests for detection dogs is still controversial [17], and it is crucial to define assessed indicators, justify their role in the DDs’ work, and establish criteria of minimum acceptance for success to be achieved. The selection of DDs based on their innate behaviors for a particular search methodology has been proposed [14], but to ensure more objectivity and reliability in the behavioral assessment tests, independent assessors in groups of two or more conducting repetitions of the tests are recommended [57].

Standardization of protocols and terminologies at an international level can optimize the breeding, selection, and performance of working dogs worldwide [24]. An international protocol is crucial to developing methods to quantify behavioral balance and identify how personality traits can influence DDs’ work success, considering some behaviors are still subjectively conceptualized according to evaluators’ expertise [16]. The high risk of anti-bomb actions demands a complex and specific process of EDD selection. Regardless of canine breed or age, multiple objective parameters to determine the dog’s qualification for detection activities must include particular exercises designed to demonstrate desirable behavioral characteristics essential in real actions of explosive detection [17,20].

## 3. Performance and Employment of EDDs

### 3.1. EDD Performance

Several aspects can be evaluated in the performance of an EDD in weapons and explosives detection, such as in a combined approach in airport security with other technologies [62]. The relationship between the handler and EDD is critical for achieving high performance, whether in training scenarios or operational reality [41], and significantly impacts the dog’s work. Therefore, a unique and exclusive handler working with a EDD may not always be the most efficient approach since a high level of dependency can decrease the dog’s autonomy and result in task failure [48]. Alternating handlers between EDDs was not perceived as a problem for high-performance dogs as long as a strict and well-defined operating protocol existed when dogs were kept active for longer periods, training progress was assessed, and potential failures were corrected [63].

The tendency to get bored after 20–30 min of exercise and changes in mood were some negative aspects detected in EDDs checking bags in US airports, which can impact their performance and increase operational costs [62]. Trained DDs can easily identify at least ten different odorants, sustaining their high detection performance for extended periods, even without additional training or reinforcement of the target odor identification [64]. In addition to the remarkable potential of DDs to track an extensive range of odorants, their impressive detection threshold (sensitivity), known as the minimum intensity of a stimulus required to generate a response [33], may also be influenced by the extent of training protocols [65]. A lack of standardized training methods and operating procedures for EDDs has remained a significant challenge in evaluating their performance over the years [4,5,24,64,65,66,67,68,69]. Several external factors can impact the performance of EDDs, environmental stimuli being one of the most significant. An example is hot weather, which can create thermal stress that considerably affects the detection activity of a dog [69,70]. Overheating harms sniffer dogs’ performance [27,37] due to their physiological and behavioral resources being diverted toward cooling the body, resulting in longer search times for positive results [8].

Panting is the primary way of cooling the body in a dog, which increases airflow through the mouth and reduces it through the nose [38,69,70]. Panting can reduce the sniffing rate in dogs because they cannot be physiologically performed simultaneously, leading to a significant loss in their detection potential [38]. Dogs’ olfactory capacity can be reduced by panting by up to 40% [71], which poses a high risk for EDDs employed in adverse hot conditions in which they need to keep their mouths closed when sniffing for prolonged periods to maintain high performance.

Short rest intervals introduced between work sessions boost dogs’ ability to engage in prolonged work, mitigating the risk of overheating [27]. Repeated exposure of animals to mild temperature elevations may trigger heat acclimation adaptations and cellular changes toward acquired thermal tolerance, which enables DDs to withstand increased levels of heat stress, conditioning and improving their performance and capacity to work under unfavorable conditions [69,70,71]. Even though security forces and police widely employ them, knowledge in the scientific literature about the effects of detection work on EDDs and how to mitigate them and improve welfare is still lacking. Considering EDDs must work at high levels of performance, excellent maintenance conditions (dog facilities and nutrition), constant veterinary assistance and checkups, taking the dog for a short walk to carry out its physiological needs (urinate and defecate) before starting work, and keeping dogs during rest intervals in air-conditioned environments are essential measures for welfare improvement and possibly contribute to better dog performance.

Individual behavioral responses such as a high level of arousal can lead to agitation, frantic searches, and difficulty moving in certain environments and controlling the dog, which can negatively impact the search efficiency and endurance of EDDs [14]. In contrast, low excitement levels can indicate a lack of interest or demotivation, which influences the dog’s motivation to conduct long searches, hampering its performance [14]. Therefore, optimal levels of excitement are vital to keeping a DD motivated and engaged and critical to maximizing its search efficiency.

Validation of the accuracy and efficiency of DDs as highly reliable tools for security policies plays a critical role in assessing investments made with public resources, ensuring that the assets are justified [17]. Some countries developed specific procedures to certify EDDs to verify acceptable performance levels and trust in them to carry out their tasks effectively. In the United Kingdom (UK), the government offers a national training and accreditation program called the National Canine Training and Accreditation Scheme—Private Security Industry (NCTAS-P), which certifies handlers and EDDs in the private security sector [72].

Law enforcement agencies in the USA rely on independent assessors, such as the Bureau of Alcohol, Tobacco, Firearms, and Explosives, for EDD certification (ATF/USA) [73]. The reliability of EDDs requires achieving hit rates above 91.6% for six different types of explosives in four or five different environments according to the North American Police Work Dog Association (NAPWDA) [67]. This benchmark is based on the principle that forensic science demands an accuracy rate of 90–95% for instrumental methods [67]. When an EDD fails to detect a real threat, it may lead to serious consequences for the dog, its handler, and others who depend on the search [48].

### 3.2. EDD Employment for a Safe World

In terms of operational employment doctrine for bombs and explosive detection, two distinct approaches have been identified. The first focuses on detecting explosive materials arranged in an isolated and inert manner (explosives), while the second assumes the possibility of an imminent artifact explosion (bombs) [73]. The US Army Field Manual for the Employment of Military Working Dogs specifies all actions in the field must be carried out in a coordinated and pre-planned manner before any intervention when bomb threats against people or property are under the responsibility of the US Army [74]. Military Working Dogs teams must only be activated to identify suspicious items in the area, following a strict protocol of operational procedures, and bomb squads will only act after locating the suspicious object and performing actions to neutralize or remove the threat. This approach, described in the manual, ensures that all measures are taken safely and with the proper precautions in order to prevent harm to the public, Military Working Dogs teams, and bomb squads [74].

The context of the explosive threat and details of the searched object determine the specific mode for EDD employment. The safety of all components is paramount, and a failure to ensure security during the search could have catastrophic consequences [75,76]. Engagement of EDDs must be carefully planned and executed under well-defined and secure search protocols [14]. A severe result was evident in the terrorist attack in Oklahoma City, USA, in 1995, where a truck filled with agricultural fertilizer, diesel oil, and other chemicals exploded, resulting in 168 deaths, hundreds of injuries, dozens of burned cars, and over three hundred damaged or destroyed nearby buildings [77].

Devasting events like this one raise doubts about the effectiveness and employment of EDDs far from their trainers, even if they have a high level of control over the dog, for three significant reasons: if the explosive charge is uncertain, a correct estimation for a security perimeter cannot be accurate; when using EDDs at a distance, buildings and architectural conditions may not enable ideal visual conditions for monitoring the movement of the dog; and the complex operational requirements of EDDs may be compromised in a sensitive activity when the concentration of explosives is unknown [14]. Even with some uncertainties regarding EDD training, further studies must be conducted to develop a trusty selection of dogs and reliable operational protocols for EDDs to avoid failures and improve performance and outcomes [48].

Harms promoted by criminal organizations and terrorists have led to a constant alert state in the combat and prevention of terror-related activities for the security of nations and their communities. Despite some limitations, EDDs have undeniable advantages, such as mobility, remarkable discriminative olfactory capacity, versatility, and solid performance in different situations. In addition to other prevention tools and human intervention, the high potential of EDDs to prevent catastrophic events can significantly mitigate the risks of destruction and loss of human lives in explosion threats, in which the potential damage is immeasurable and irreparable. Therefore, integrating EDDs into security systems requires a sensitive approach to selecting dogs, considering situations in which they will be employed, and standardizing protocols among security forces.

While some countries have implemented their certification systems for EDDs, there is a need to standardize international models with impartial and independent evaluations conducted by specialized-in-law inspectors, including a minimum number of risk scenarios that closely replicate real-life situations.

## 4. Conclusions

In conclusion, this review showed numerous advantages in EDDs’ employment, their risks, fragilities, and the need for periodic reevaluations of EDDs’ performance to ensure minimum safe standards and management in usually highly life-threatening situations. Also, this review addressed particular aspects of EDD training and work, requiring a high level of sensitivity and frequent contact with hazardous situations directly related to real explosions. Procedures for working with EDDs are unique and distinct from other forms of detection, including behavioral and physical features evaluation during selection, which is fundamental to their employment in explosives detection. The cognitive and emotional capacities of EDDs have to be aligned in training programs, including factors such as repetition, reinforcement, and the surrounding environment. In the selection of EDDs, high performance and essential characteristics such as trainability, self-confidence, focus, resilience, olfactory acuity, precision, and welfare must be continually refined. The certification system for EDDs is fundamental in order to standardize the work of dogs, and it must be impartially and independently conducted by specialized inspectors within the law, ensuring that EDDs can be employed and perform their best, even in adverse conditions. The use of EDDs at high levels of performance in conjunction with explosive detection technologies must be taken into account as a formidable barrier to restrain terrorist attacks and illegal trade with explosives.

## Data Availability

Not applicable.
